# Supplementary material for: Amino acid gas phase circular dichroism and implications for the origin of biomolecular asymmetry
Source: Nat Commun. 2022 Jan 26;13:502. doi: 10.1038/s41467-022-28184-0 (PMC8792022; doi:10.1038/s41467-022-28184-0)
Supplement: Supplementary file 1 — Supplementary Information [file 41467_2022_28184_MOESM1_ESM.pdf]

SUPPLEMENTARY INFORMATION

Amino acid gas phase circular dichroism and implications for the origin of biomolecular asymmetry

Cornelia Meinert[1*], Adrien D. Garcia[1‡], Jérémie Topin[1‡], Nykola C. Jones[2], Mira Diekmann[3], Robert Berger[3], Laurent Nahon[4], Søren V. Hoffmann[2] & Uwe J. Meierhenrich[1*]

[‡]These authors contributed equally to this work.

correspondence to: cornelia.meinert@univ-cotedazur.fr, uwe.meierhenrich@univ-cotedazur.fr

This file contains Supplementary Material, a detailed description of results not reported in the main text and additional analyses. It contains Supplementary Figs 1–15 and Supplementary Tables 1–3.

Supplementary Methods

Chemicals page 2

Gas-Phase Circular Dichroism & Anisotropy measurements page 2

Chromatography measurements page 3

Computational details page 4

Supplementary Notes

Supplementary Note 1: Gas phase CD and anisotropy spectra of amino acids page 5

- CD, absorption, and anisotropy spectra – *alanine*
- Normalized CD and anisotropy spectra – *2-aminobutyric acid*
- Normalized CD and anisotropy spectra – *proline*
- Normalized CD and anisotropy spectra – *valine*
- Normalized CD and anisotropy spectra – *isovaline*
- Normalized CD and anisotropy spectra – *norvaline*
- Normalized CD and anisotropy spectra – *leucine*

Supplementary Note 2: Shape dependence of CD spectra on gas pressure page 11

Supplementary Note 3: Thermal degradation products of amino acids page 12

Supplementary Note 4: Quantum chemical calculations page 14

Supplementary Note 5: Polymerization test of alanine at elevated temperature page 18

Supplementary References page 20

Supplementary Methods

Chemicals

Enantiopure standards of the following amino acids were purchased from Sigma-Aldrich (Merck, Germany) and were of the following purity: L-alanine (≥99.5 % purity), D-alanine (≥98 % purity), L-2-aminobutyric acid (99 % purity), D-2-aminobutyric acid (98 % purity, 98 %*ee*), D-proline (≥99 %, 98 %*ee*), L-valine (≥99.5 % purity), D-valine (≥99 %), D-norvaline (99 % purity, 98 %*ee*), L-leucine (≥99.5 % purity), D-leucine (99 % purity, 97 %*ee*), L-phenylalanine (≥99 %), and L-alanyl-L-alanine (≥98 % purity). L-Proline (99 % purity), L-norvaline (99 % purity), and L-serine (99 % purity) were purchased from Alfa Aesar and L-isovaline (99 %, >99 %*ee*), and D-isovaline (99 % purity) from Across Organics. The cyclodipeptide cyclo(-Ala-Ala) (>99 % purity) was purchased from Bachem. 1*R*-(+) camphor (98 %) used for calibration purposes of the gas cell prior recording amino acid circular dichroism spectra was purchased from Sigma Aldrich (Germany).

Gas-Phase Circular Dichroism & Anisotropy Measurements

Historically, gas phase CD measurements have mostly been limited to volatile molecules, such as e.g. carvone[1,2], limonene[3], and camphor[4]. Common for all these compounds is that they exhibit a sufficiently high vapour pressure to allow measurements in a relative short pathlength cell of 1 cm operated near room temperature, although longer gas cells have been used to measure the CD of substituted 4-methylcyclohexylidenes[5]. More recently, ion spectroscopy was used to measure gas phase CD spectra of DNA oligonucleotides[6] overcoming thermal instabilities by using an electrospray source that on the other hand, however, prevented the measurement of DNA strands in their neutral form. So far, gas phase CD measurements of neutral amino acids have not been reported. The very low vapor pressure of amino acids implies that in order to achieve adequate signals, a combination of elevated temperatures, up to 200 °C, and a long pathlength cell are required.

To overcome these challenges linked to amino acid gas phase measurements, we recorded circular dichroism spectra $CD(\lambda)$, defined as $\Delta\varepsilon = \varepsilon_L - \varepsilon_R$; where ε_L and ε_R are extinction coefficients of left- and right-CPL, respectively; anisotropy spectra $g(\lambda)$, and absorption spectra $A(\lambda)$ of seven pairs of amino acid enantiomers at the ASTRID2 synchrotron radiation source, Aarhus University, Denmark. We have developed a gas cell that is temperature and pressure controlled. The cell is mounted on the AU-CD beam line, where CD measurements can be performed in the wavelength range 130–500 nm.[7,8] The cell consists of a stainless-steel tube, 500 mm in length with an 18 mm inner diameter that can be filled, under vacuum, with a specific chiral analyte. Using a synchrotron radiation source, with much lower beam divergence than found in conventional lamp-based CD spectrometer, allowed for the operation of such a long gas cell. CaF_2 windows enclose the extremities of the gas cell, which has a photoelastic modulator (PEM) to produce the circularly polarised light at one end and a photomultiplier tube (PMT) detector at the other. The section between the PEM and the entrance CaF_2 window is purged with nitrogen, while the section on the exit of the cell to the detector is kept under vacuum using a dry scroll pump, to remove absorption due to oxygen and water.

The pressure inside the gas cell is monitored using a type 631D Baratron heated manometer (MKS instruments) which is maintained at a temperature of 200 °C, while the cell is evacuated using a Varian V70 turbo pump. Two all-metal valves are mounted on the main gas cell tube: one connects, via a flexible hose, to the turbo pump, while the other valve connects to the heated sample reservoir. This allows the pumping speed and sample inlet rate to the gas cell to be adjusted. Heating tapes and cartridge heaters were used to heat the entire gas cell up to the required temperature using PID computer control, with a maximum possible temperature of 200 °C. The temperature is monitored,

and locally controlled, at nine different positions around the gas cell assembly. Differential heating of the windows to 5–10 °C higher temperatures compared to the tube avoids local condensation of analytes. The gas cell has been designed so that it can quickly be taken apart, cleaned, parts replaced if necessary and reassembled. Cleaning was typically performed after each measurement campaign at the AU-CD beam line with an average duration of one week. Residual amino acids in e. g. one of the all-metal valves, was a good indication of cold spots from suboptimal heat distribution. Heating was added and re-arranged to ensure cold spot free operation. To protect the turbo pump from condensed amino acid powder, the inevitable temperature gradient from high temperatures on the gas cell to room temperature at the pump was arranged to occur along the flexible hose, with a water-cooled Cu block placed in the vacuum system between the hose and the turbo pump. After each cleaning, the entire gas cell was baked off-line under vacuum at 125 °C for a week, to ensure minimal residual water pressure from the clean gas cell.

Calculations of the vapour pressure, $p = f(T)$, of the amino acids showed that the gas cell would provide sufficient molecular gas phase density to allow for gas phase CD and anisotropy spectroscopy. Based on our experience of gas phase photoabsorption of small molecules[9], we expected to require a minimum amino acid pressure of approximately p = 0.03 mbar in a gas cell with an optical path length of d = 500 mm. For alanine and other amino acids, vapour pressures have previously been recorded as a function of the temperature.[10] Alanine reaches a measurement pressure of p = 0.03 mbar at T = 160 °C. We know from previous sublimation experiments of alanine and other amino acids[11,12] that this temperature can be achieved without fully decomposing the amino acid analytes. Jochims *et al.* showed by VUV-mass spectrometry on several amino-acids, including α-alanine, aminoisobutyric acid, and valine, that in this temperature range decomposition can be avoided.[13] Also Blanco *et al.* performed gas phase microwave spectroscopy on alanine and other amino acids at a temperature of 225 °C, showing that such temperatures are achievable in the gas phase without complete thermal destruction of the analytes.[14]

Typically, less than 1 g of a given amino acid enantiomer was placed into a glass container (Ø12 mm, length 15 mm) and inserted into the sample reservoir heater. This amount of sample proved to be enough for several measurement runs including different temperature scans. Generally, all amino acids tested in our study are characterized by relatively high melting points and low vapour pressures. We therefore started to record the absorbance and CD spectra of each amino acid at T = 160 °C and gradually increased the temperature until sufficient vapour pressures with optimal optical densities were reached. The maximum time to record one circular dichroism and anisotropy spectrum under optimal conditions is 1 h. In the cases where the signal to noise (S/N) values were low, this time had to be increased by a factor of 5 to 10. For samples with very low vapour pressure and too low S/N values the temperature in the gas cell was increased to a maximum of T_{max} = 190 °C.

The gas cell was typically operated under flow conditions, where the valves to the sample and the turbo pump were partially open, to ensure a continuous renewal of the gas in the cell. This was done to avoid a build-up of the products of amino acid decomposition, see section S8 for further details.

Chromatography measurements

Enantioselective multidimensional gas chromatography–time-of-flight mass spectrometry

The GC×GC–TOF-MS Pegasus IV D system from LECO comprised a time-of-flight mass spectrometer operated at a storage rate of 100 Hz, with a 50–400 amu mass range and a detector voltage of 1.45 kV. Data were processed using the LECO Corp. ChromaTOF™ software. The source and transfer temperature were kept at 230 and 240 °C, respectively. The column set consisted of a Chirasil-L-Val column (25 m × 0.25 mm inner diameter, 0.12 mm film thickness) in the first-dimension modulator-coupled to a DB Wax secondary column (1.5 m × 0.1 mm inner diameter, 0.1 mm film thickness). Helium was used as carrier gas at a constant flow of $\bar{u}$ = 1 mL min^{-1}. Sample volumes (1 µL) were injected in splitless mode at an injector temperature of 230 °C. The GC primary oven was operated as follows: 40 °C (1 min), warm up to 80 °C (10 min) at 10 °C min^{-1}, and warm up to 190 °C (15 min) at 4 °C min^{-1}. The secondary oven used the same temperature program with a constant temperature offset of 20 °C. A modulation period of P_M = 5 s was applied.

Our standardized derivatisation protocol for amino acids follows. Each amino acid sample was dissolved in 0.1 M HCl and an aliquot of 50 µL was transferred into a reaction vial, to which 25 µL of a heptafluoro-1-butanol/pyridine mixture (3:1, v/v) was added. Ethyl chloroformate (5 µL) was added to this solution. The vials were capped tightly and shaken vigorously for 10 seconds to form *N*-ethoxy-carbonylheptafluorobutylester (ECHFBE) derivatives. Finally, chloroform (50 µL) containing methyl laurate at 10^{-5} M as an internal standard was added, and the vials were shaken again for extraction of the amino acid ECHFBE derivatives into the organic phase. The organic phase was withdrawn and transferred into 1 mL GC vials equipped with 100 µL inserts for enantioselective GC×GC-TOFMS analysis.

Liquid chromatography measurements

The HPLC Agilent 1200 system was composed of a quaternary pump (Agilent G1311 A) equipped with a Diode Array Detector (DAD Agilent G1315D) and an ELSD (Evaporative Light Scattering Detector). A Luna C18 column (Phenomenex, 150×4.6 mm, 5 µm) was used at 25 °C with an injection volume set at 10 µL and a flow rate set at 1.0 mL min^{-1}. The HPLC was used in isocratic mode with 95 % chromatography grade water (A) (acidified with 1 % formic acid) and 5 % methanol (B) during 40 min.

Computational details

Time-dependent density functional theory calculations (TD-DFT)

A thorough conformational search was achieved by a biased molecular dynamics simulation following an Umbrella Sampling protocol.[15] A single alanine residue was described in the neutral state using the ff14SB force field.[16] Two dihedral angles were constrained during the simulation to allow for an exhaustive sampling of all rotational angles. The clustering of the trajectory resulted in an initial set of 20 alanine conformers. Two more conformations were added by manually rotating the hydrogen from the amine function.

All selected structures were refined with Gaussian16[17] using a combination of different methods and a large basis set quadruple-ζ with polarization and diffuse functions: aug-cc-pvQZ.[18] A single-point energy calculation with zero-point corrected energies including frequency calculations was done to verify their nature of true minima. Three methods were evaluated: CAM-B3LYP[19], ωB97X-D[20] and MO6-2X[21]. Finally, seven low energy conformers were further considered for calculations. TD-DFT calculations were done on the seven optimized structures using the three previous functionals combined with the aug-cc-pvQZ basis set. 200 excited states were calculated for each optimized conformer.

Natural transition orbitals (NTO)

To obtain a compact orbital representation of the electronic transition excitation, the ordinary orbitals were transformed.[22] This calculation was applied to conformer 4 for which we observed a negative CD band at 230 nm. The computed natural transition orbitals (NTOs) for this state lead to a qualitative description of the electronic excitations.

Coupled cluster calculations

Csaszar's[23] thorough study of alanine conformers was used as a starting point, from which all reported thirteen local minimum structures with their corresponding harmonic vibrational wavenumbers were obtained and subsequently Boltzmann-weighted. A total of nine alanine conformers comprising conformers **I**, **IIA/B**, **IIIA/B**, **IVA/B**, and **VA/B** – all in (*S*)-configuration – were included in the computation of the CD spectrum.

All 13 conformer structures were energy optimized with the program package Molpro[24,25,26] on the df-CCSD(T)-F12 level with the aug-cc-pVDZ-F12 basis set, specifically with the F12b method and the 3*C(FIX, HY1) ansatz as implemented in Molpro. Harmonic vibrational wavenumbers were calculated on the same level. Relative energies were determined by single point calculation with CCSD(T)-F12 and aug-cc-pVTZ-F12 basis set on the structures optimized with the double zeta basis set. Electronic excitation energies and intensities of the CD and the one-photon absorption spectra were calculated with the program package Turbomole[27] with the RI-CC2 method and the aug-cc-pVQZ basis set.

Supplementary Notes

Supplementary Note 1 : Gas phase circular dichroism and anisotropy spectra of amino acids

Although desirable, the measured pressure of the gas in the cell was not a direct measure of the amino acid gas density. Other contaminants such as water vapour contribute to the overall pressure. Additionally, the partial pressure component of e. g. water will depend on sample temperature as well as on the flow rates under which the gas cell was operated. Therefore, the CD spectrum of an amino acid could not be scaled directly with the cell pressure during measurements and the absolute CD signal (corrected for gas density and pathlength) could not be calculated. Thus, the CD spectra shown are in units of ellipticity measured in millidegrees (mdeg). CD spectra were mildly smoothed with a Savitzky-Golay filter using a second-order polynomial fit and a 7-point window. To provide the best representative CD spectrum of an amino acid, spectra were measured at different cell temperatures and various gas flow rates. For a majority of individually recorded spectra for each individual amino acid enantiomer, the CD signal could be normalized to yield spectra of similar shape and magnitude by scaling with a factor determined by scaling their corresponding absorbance spectra, $A(\lambda)$, in the 170–230 nm spectral range. The conditions for good scalability were when the temperature was high enough for sufficient amino acid gas pressure while the gas flow rate was sufficient to ensure low levels of contaminants. For alanine, the average of a set of CD spectra for each of the two enantiomers are shown in **Supplementary Fig. 1**, as well as the final spectra normalized to absorbance. The process yielded two nicely mirrored CD spectra of the L- and D-form of alanine. The same approach was applied to all other amino acid CD spectra, **Supplementary Figs. 2**–**7**, where only the normalized CD spectra are shown. Finally, we note that this normalization process has no influence on the anisotropy spectra, $g(\lambda) = CD(\lambda)/A(\lambda)$, as they are independent of any scaling factor applied to both absorbance and CD spectra.

Alanine

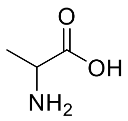

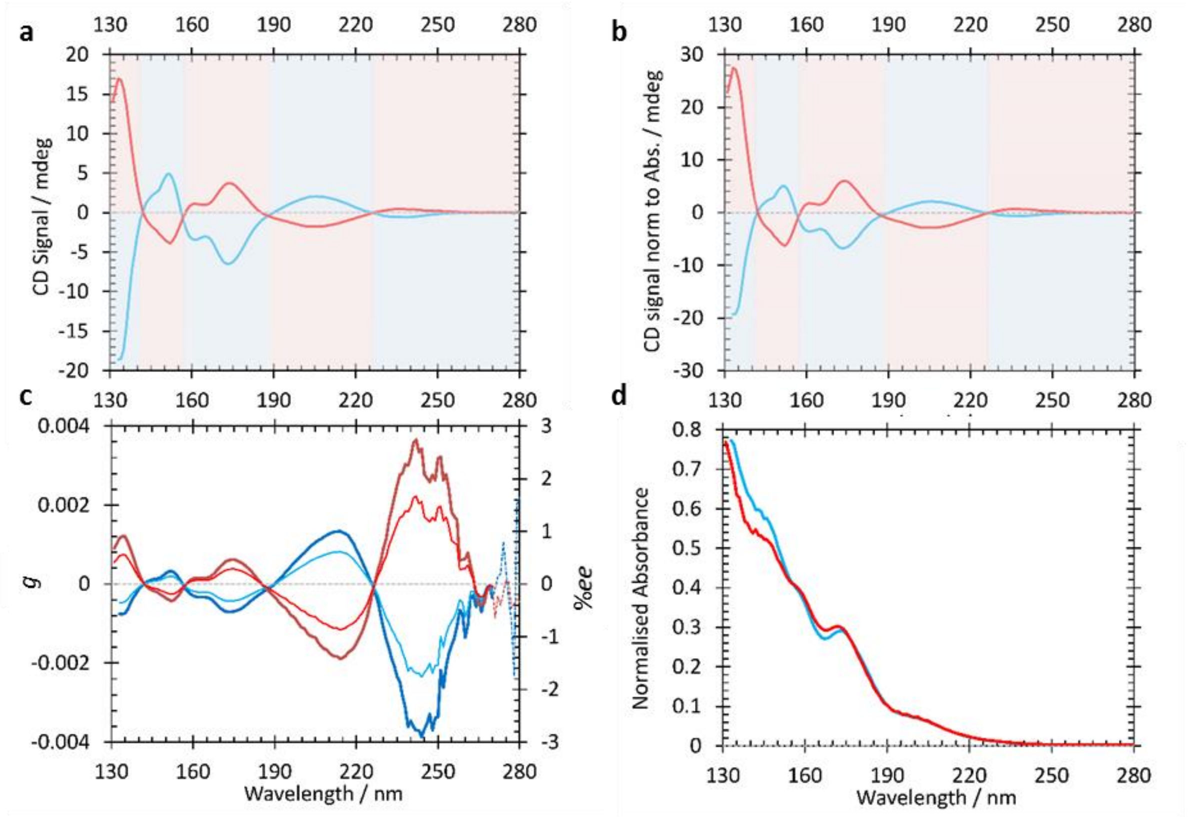

Supplementary Figure 1: CD, absorption, and anisotropy spectra of L-alanine (blue) and D-alanine (red). a, non-normalized CD spectra. **b**, CD spectra scaled according to their absorbance spectra. **c,** Anisotropy spectra (thick lines) and the enantiomeric excess (%*ee*, thin lines) obtainable via photolysis of a racemic alanine sample according to Meinert *et al.*[11] **d,** The absorbance curves of the two enantiomers after normalization in the range 230–180 nm.

2-Aminobutyric acid

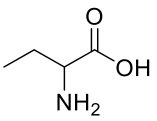

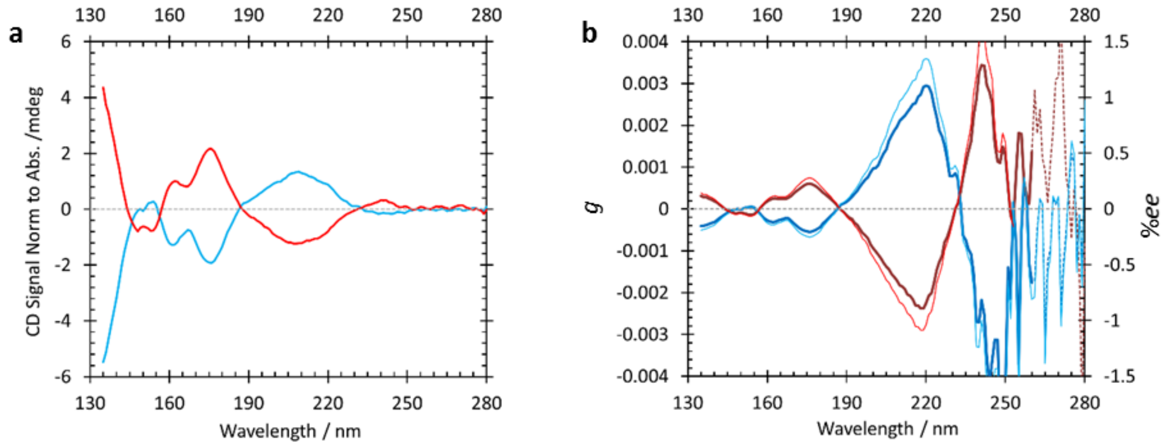

Supplementary Figure 2: **Normalized CD and anisotropy spectra of L-2-aminobutyric acid (blue) and D-2-aminobutyric acid (red). a,** CD spectra normalized according to their absorbance spectra. **b,** Anisotropy spectra (thick lines) and the enantiomeric excess (%*ee*, thin lines) obtainable via photolysis of a racemic 2-aminobutyric acid sample according to Meinert *et al.*[11]

Proline

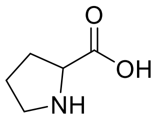

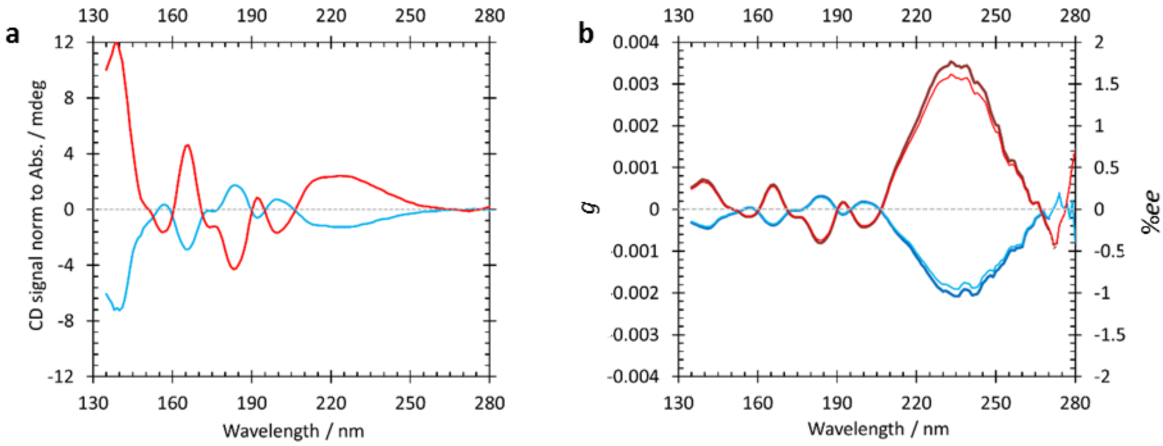

Supplementary Figure 3: Normalized CD and anisotropy spectra of L-proline (blue) and D-proline (red). a, CD spectra normalized according to their absorbance spectra. **b,** Anisotropy spectra (thick lines) and the enantiomeric excess (%*ee*, thin lines) obtainable via photolysis of a racemic proline sample according to Meinert *et al.*[11]

Despite the relatively high purity of both proline standards (L-proline 99 % purity, unknown %*ee,* T_{melt} = 221 °C, Alfa Aesar – A10199, LOT: 10202851; D-proline ≥ 99%, 98% *ee,* T_{melt} = 223 °C, Sigma Aldrich – 858919, LOT: 10701PHV) and very similar absorbance curves for the L- and D-enantiomers, the CD and anisotropy spectra of the L-proline enantiomer differ approximately by a factor 2 compared with the D-enantiomer. As the enantiopurity of L-proline is not stated, we expect that the L-enantiomer is less enantiomerically pure leading to the overall lower intensity of the CD and *g* bands.

Valine

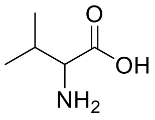

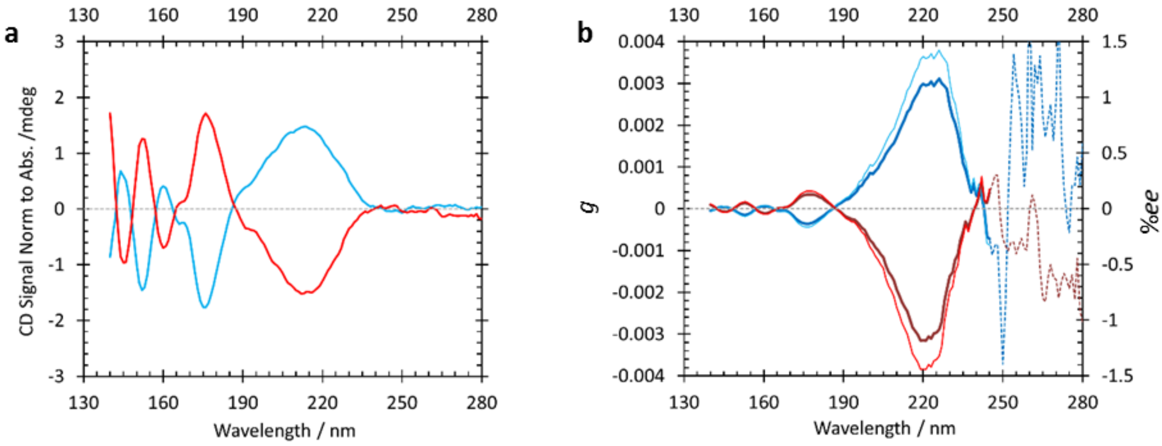

Supplementary Figure 4: Normalized CD and anisotropy spectra of L-valine (blue) and D-valine (red). a, CD spectra normalized according to their absorbance spectra. **b,** Anisotropy spectra (thick lines) and the enantiomeric excess (%*ee*, thin lines) obtainable via photolysis of a racemic valine sample according to Meinert *et al.*[11]

Isovaline

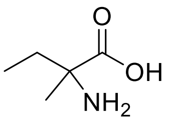

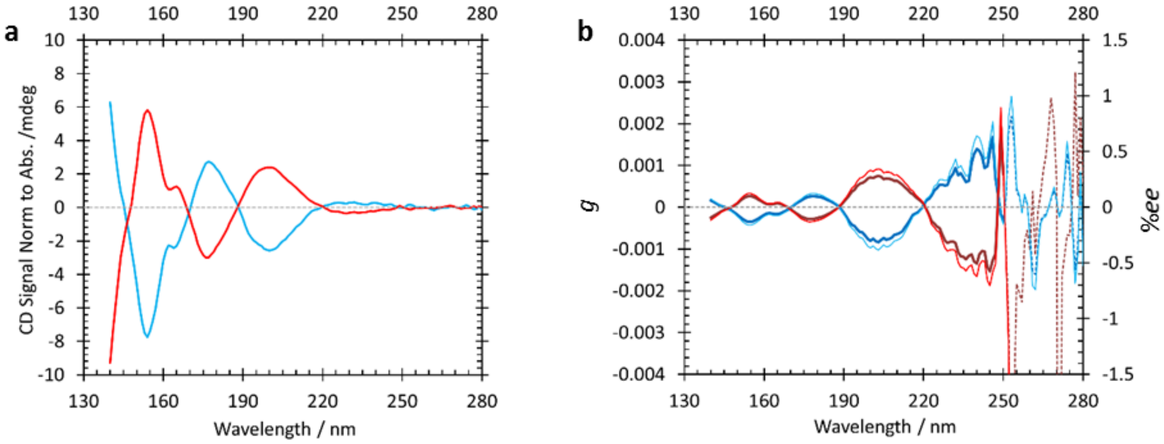

Supplementary Figure 5: Normalized CD and anisotropy spectra of L-isovaline (blue) and D-isovaline (red). a, CD spectra normalized according to their absorbance spectra. **b,** Anisotropy spectra (thick lines) and the enantiomeric excess (%*ee*, thin lines) obtainable via photolysis of a racemic isovaline sample according to Meinert *et al.*[11]

Norvaline

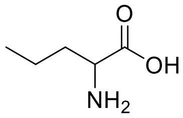

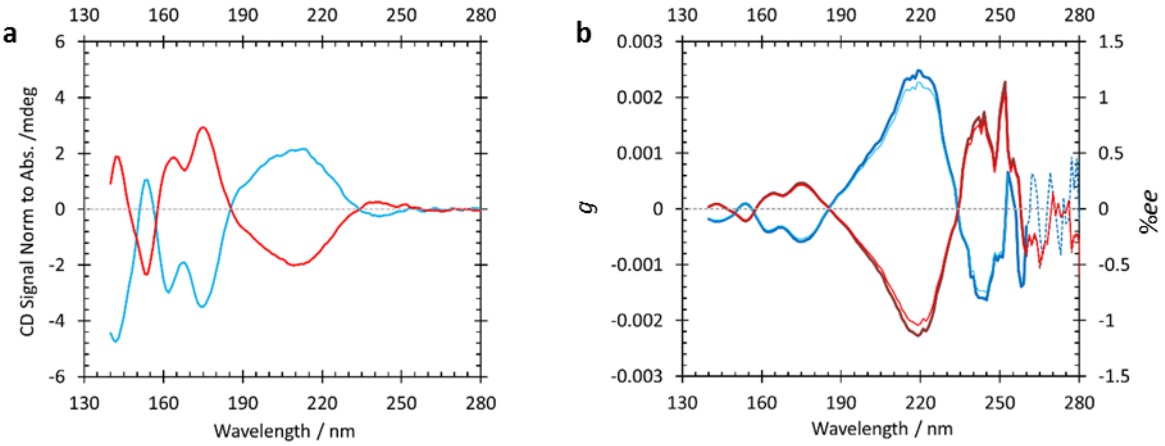

Supplementary Figure 6: Normalized CD and anisotropy spectra of L-norvaline (blue) and D-norvaline (red). a, CD spectra normalized according to their absorbance spectra. **b,** Anisotropy spectra (thick lines) and the enantiomeric excess (%*ee*, thin lines) obtainable via photolysis of a racemic norvaline sample according to Meinert *et al.*[11]

Leucine

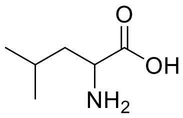

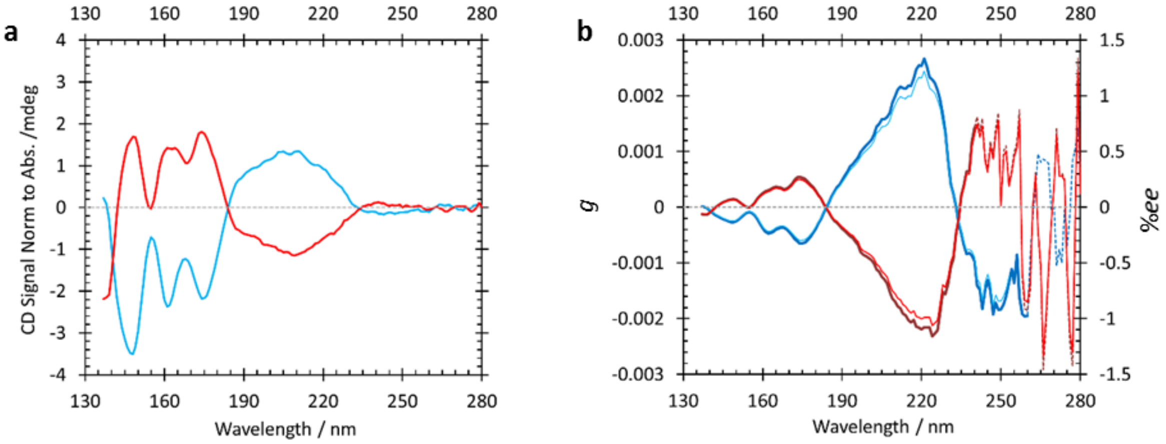

Supplementary Figure 7: Normalized CD and anisotropy spectra of L-leucine (blue) and D-leucine (red). a, CD spectra normalized according to their absorbance spectra. **b,** Anisotropy spectra (thick lines) and the enantiomeric excess (%*ee*, thin lines) obtainable via photolysis of a racemic leucine sample according to Meinert *et al.*[11]

Supplementary Table 1: Experimental electronic excitation energies (Δ*E*), electronic circular dichroism (Δ*ε*), anisotropy values *g* and inducible %*ee* values of the most intense gas phase dichroic transitions of amino acids. Inducible %*ee* values are given for an extent of reaction *ξ* = 0.9999 and *right*-circularly polarized light.

	state	transition	**Δ*E* (eV)**	***λ* (nm)**	**Δ*ε* (mdeg)**	**Δ*E* (eV)**	***λ* (nm)**	***g* (×10^-3^)**	***ee* (%)**
**L-Alanine**	^{1}S (1)	$n_O \rightarrow \pi^*_{CO}$	5.19	239	-0.63	5.08	244	-3.87	-1.77
	^{1}S (2)	$n_N \rightarrow 3s$	5.99	207	2.07	5.79	214	1.34	0.61
	^{1}S (3)	$n_O \rightarrow 3s$	7.17	173	-6.76	7.17	173	-0.70	-0.32
	^{1}S (4)	$n_N \rightarrow 3p$	7.70	161	-3.61	7.61	163	-0.34	-0.16
	^{1}S (5)	$n_O \rightarrow 3p$	8.21	151	5.06	8.16	152	0.33	0.15
**L-2-Aminobutyric acid**	^{1}S (1)	$n_O \rightarrow \pi^*_{CO}$	5.08	244	-0.16	5.08	244	-4.17	-1.90
	^{1}S (2)	$n_N \rightarrow 3s$	5.93	209	1.34	5.64	220	2.95	1.35
	^{1}S (3)	$n_O \rightarrow 3s$	7.05	176	-1.93	7.05	176	-0.55	-0.25
	^{1}S (4)	$n_N \rightarrow 3p$	7.70	161	-1.28	7.65	162	-0.31	-0.14
	^{1}S (5)	$n_O \rightarrow 3p$	8.05	154	0.27	8.05	154	0.05	0.02
	^{1}S (6)	$\pi_{C=O} \rightarrow 3s$	8.32	149	0.01	8.32	149	0.002	7×10^{-4}
**D-Proline¶**	^{1}S (1)	$n_O \rightarrow \pi^*_{CO}$	5.56	223	2.40	5.32	233	3.54	1.62
	^{1}S (2)	$n_N \rightarrow 3s$	6.23	199	-1.66	6.20	200	-0.43	-0.20
	^{1}S (3)	$n_O \rightarrow 3s$	6.46	192	0.84	6.46	192	0.18	0.08
	^{1}S (4)	$n_N \rightarrow 3p$	6.78	183	-4.29	6.74	184	-0.80	-0.37
	^{1}S (5)	$n_N \rightarrow 3p$	7.13	174	-1.28	7.13	174	-0.18	-0.08
	^{1}S (6)	$n_N \rightarrow 3p$	7.47	166	4.62	7.47	166	0.60	0.27
	^{1}S (7)	$n_O \rightarrow 3p$	7.95	156	-1.63	7.95	156	-0.18	-0.08
	^{1}S (8)	$\pi_{C=O} \rightarrow 3s$	8.92	139	12.08	8.92	139	0.72	0.33
**L-Valine**	^{1}S (1)	$n_O \rightarrow \pi^*_{CO}$	5.85	213	1.47	5.49	226	3.12	1.42
	^{1}S (2)	$n_O \rightarrow 3s$	7.04	176	-1.76	7.04	176	-0.36	-0.17
	^{1}S (3)	$n_N \rightarrow 3p$	7.47	166	-0.27	7.47	166	-0.05	-0.02
	^{1}S (4)	$n_N \rightarrow 3p$	7.75	160	0.41	7.75	160	0.06	0.03
	^{1}S (5)	$n_O \rightarrow 3p$	8.16	152	-1.46	8.10	153	-0.15	-0.07
	^{1}S (6)	$\pi_{C=O} \rightarrow 3s$	8.61	144	0.68	8.61	144	0.05	0.02
**L-Norvaline**	^{1}S (1)	$n_O \rightarrow \pi^*_{CO}$	5.14	241	-0.25	5.06	245	-1.63	-0.75
	^{1}S (2)	$n_N \rightarrow 3s$	5.82	213	2.16	5.66	219	2.49	1.14
	^{1}S (3)	$n_O \rightarrow 3s$	7.13	174	-3.49	7.08	175	-0.58	-0.27
	^{1}S (4)	$n_N \rightarrow 3p$	7.65	162	-2.99	7.65	162	-0.42	-0.19
	^{1}S (5)	$n_O \rightarrow 3p$	8.10	153	1.47	8.05	154	0.10	0.05
	^{1}S (6)	$\pi_{C=O} \rightarrow 3s$	8.73	142	-4.75	8.67	143	-0.22	-0.10
**L-Isovaline**	^{1}S (1)	$n_O \rightarrow \pi^*_{CO}$	5.34	232	0.32	5.04	246	1.68	0.77
	^{1}S (2)	$n_N \rightarrow 3s$	6.20	200	-2.57	6.11	203	-0.83	-0.38
	^{1}S (3)	$n_O \rightarrow 3s$	7.00	177	2.74	6.93	179	0.27	0.12
	^{1}S (4)	$n_N \rightarrow 3p$	7.56	164	-2.44	7.56	164	-0.17	-0.08
	^{1}S (5)	$n_O \rightarrow 3p$	8.05	154	-7.75	8.05	154	-0.35	-0.16
**L-Leucine**	^{1}S (1)	$n_O \rightarrow \pi^*_{CO}$	5.10	243	-0.17	5.02	247	-1.92	-0.88
	^{1}S (2)	$n_N \rightarrow 3s$	5.90	210	1.34	5.61	221	2.67	1.22
	^{1}S (3)	$n_O \rightarrow 3s$	7.13	174	-2.17	7.13	174	-0.65	-0.30
	^{1}S (4)	$n_N \rightarrow 3p$	7.70	161	-2.37	7.65	162	-0.47	-0.22
	^{1}S (5)	$\pi_{C=O} \rightarrow 3s$	8.38	148	-3.51	8.32	149	-0.27	-0.12

¶ Normalized data based on absorbance of purer D-enantiomer.

Supplementary Note 2: Shape dependence of CD spectra on gas pressure

The CD and corresponding absorbance spectra of both the D- and L-enantiomer of alanine measured at various gas pressures are shown in **Supplementary Fig. 8a** and **8b**, respectively. Although the signal strengths obviously depend on the gas pressure, there is no apparent shape dependence on the pressure. To further elucidate if there is any shape change with sample pressure, the absorbance spectra have been scaled to the same values in the wavelength range 170–190 nm (**Supplementary Fig. 8d**), and the corresponding scaling factors are applied to the CD spectra as shown in **Supplementary Fig. 8c**. The scaling of the absorbance spectra merges all individual spectra together for wavelengths above 160 nm. The variation observed below 160 nm is attributed to the lower level of light intensity combined with the higher absorbance in this wavelength range, as well as to some extent from the nitrogen purge gas that is present in the beam path outside the gas cell. Performing the scaling to the CD spectra brings them close to each other in a small range of overall signal magnitudes, but also highlights that no shape change is visible in the collection of a total of 15 different spectra. Therefore, within the range of gas pressures obtainable in the experimental set-up, all below 0.1 mbar, we do not observe any gas pressure induced shape change in the CD spectra.

Within the various datasets presented below, there are some spectra which upon close inspection show a contribution from ammonia in the absorbance spectrum which will affect the results of the normalisation of the CD spectra and hence the anisotropy spectra (see below for more discussions on ammonia). However, despite this, there is good correspondence of all the normalised CD curves.

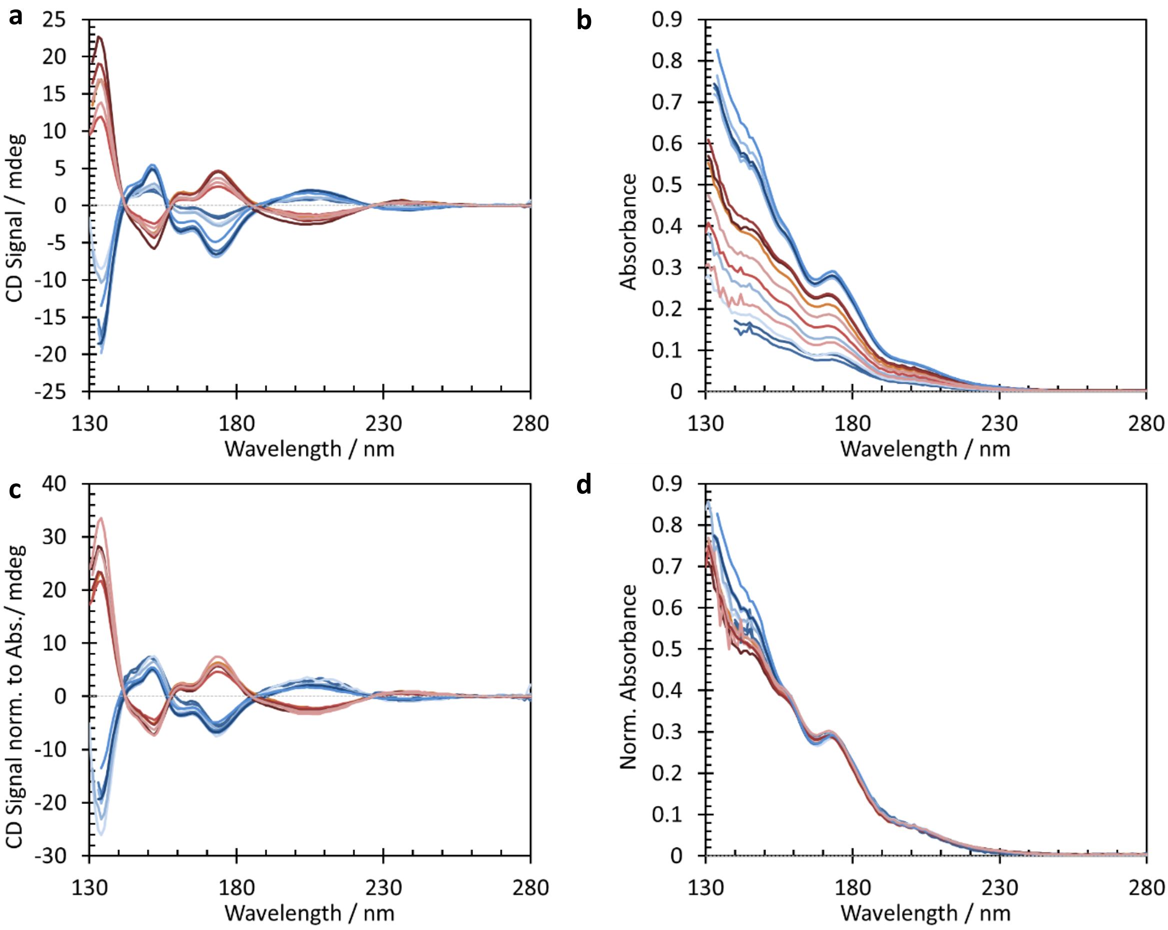

Supplementary Figure 8: Alanine CD shape dependence on different gas cell pressures. a, CD and **b**, absorbance spectra of L-alanine (blue) and D-alanine (red) measured at different gas pressures. **c**, CD spectra of L-alanine (blue) and D-alanine (red) normalized according to their **d**, absorbance spectra normalized in the wavelength range 170–190 nm.

Supplementary Note 3: Thermal degradation products of amino acids

We observed non-photon triggered formation of ammonia in the gas cell via clear indications of NH_3 absorption features forming over time under closed gas cell conditions, when the valves to the sample and turbo pump were closed after the inlet of amino acid gas. The rate of evolution of ammonia during about 30 minutes was similar irrespective of whether the target amino acid was constantly irradiated with photons or if the sample was left un-irradiated in the cell at elevated temperatures. Thus, besides photolysis the amino acid analytes undergo thermolysis. For the control of thermolysis we recorded the gas phase spectrum of ammonia (**Supplementary Fig. 9**). A second thermal degradation product of all amino acids is water (**Supplementary Fig. 10**). Additionally, the amino acid powder contains water and due to its very high vapour pressure compared to the amino acid it inevitably will be part of the total gas pressure in the cell.

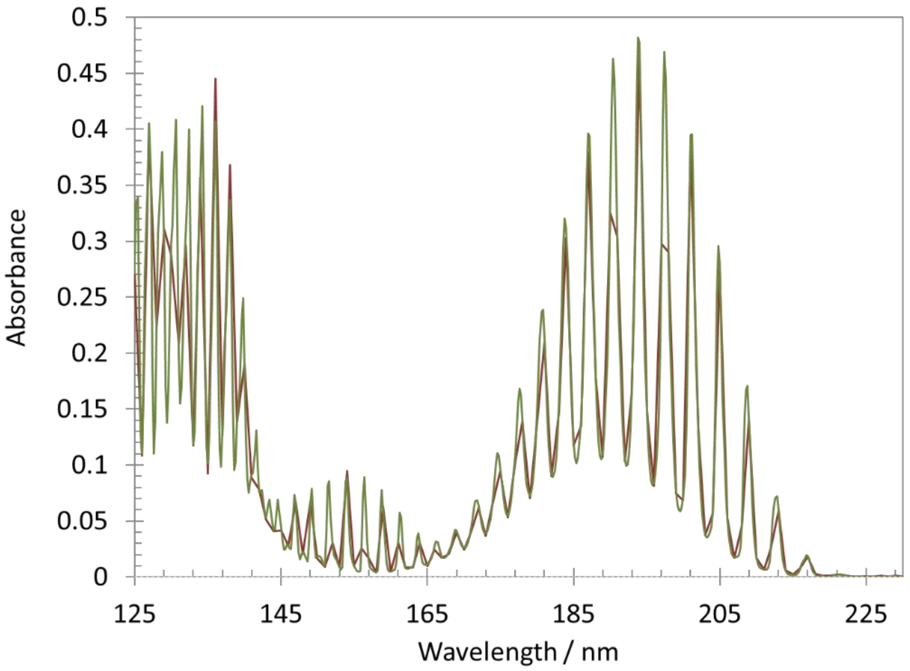

Supplementary Figure 9: High-resolution absorbance spectrum of ammonia measured with 0.2 mm (green) and 1.0 mm (brown) steps, recorded using the gas cell on the AU-CD beam line.

Contamination generally results in a noisier anisotropy spectrum and the effect of any contaminant is to lower the anisotropy spectrum, since the non-chiral contaminant absorption is included when calculating the anisotropy $g = CD/A$. Therefore, we underline that the data presented here represent a lower limit for the anisotropy values. However, the contamination from thermolysis was kept to a minimum by operating the gas cell under flow conditions, see section S2. To minimize the level of water coming from the sample itself, each sample was slowly heated to 80 °C and pumped on for at least 2 hours prior to each anisotropy measurement. Further, once the gas cell reached the operating temperature and prior to establishing the flow condition for measurement, the sample valve was briefly fully opened two to three times to pump away water build-up in the sample container. These steps ensured the lowest possible water impurities during measurements.

The effect of degradation products on the spectra is most noticeable in the anisotropy spectrum of isovaline (**Supplementary Fig. 5b**). The spectrum clearly shows fine structure in the 190–210 nm wavelength range which is absent in the CD spectrum (**Supplementary Fig. 5a**). It originates from ammonia features in the absorbance spectrum used to obtain the anisotropy spectrum.

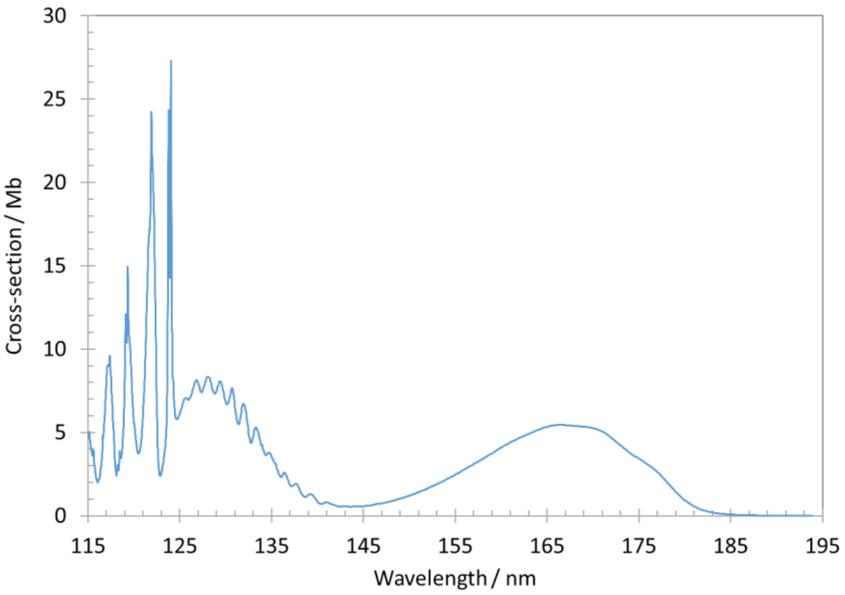

Supplementary Figure 10: High resolution absorption spectrum of water[28] measured on the AU-UV beam line.

A scaled ammonia spectrum, shown in **Supplementary Fig. 9**, may be subtracted from the measured absorbance spectrum of isovaline to produce a spectrum free of ammonia features, see **Supplementary Fig. 11a**. Using this as the absorbance spectrum to calculate the anisotropy spectrum of isovaline removes the effect of ammonia on the magnitude of the anisotropy in the 190–210 nm wavelength range as shown in **Supplementary Fig. 11b**. The effect is modest in this wavelength range, with local variations in the anisotropy values of up to 10 %, but no increase in the maximum anisotropy of the band.

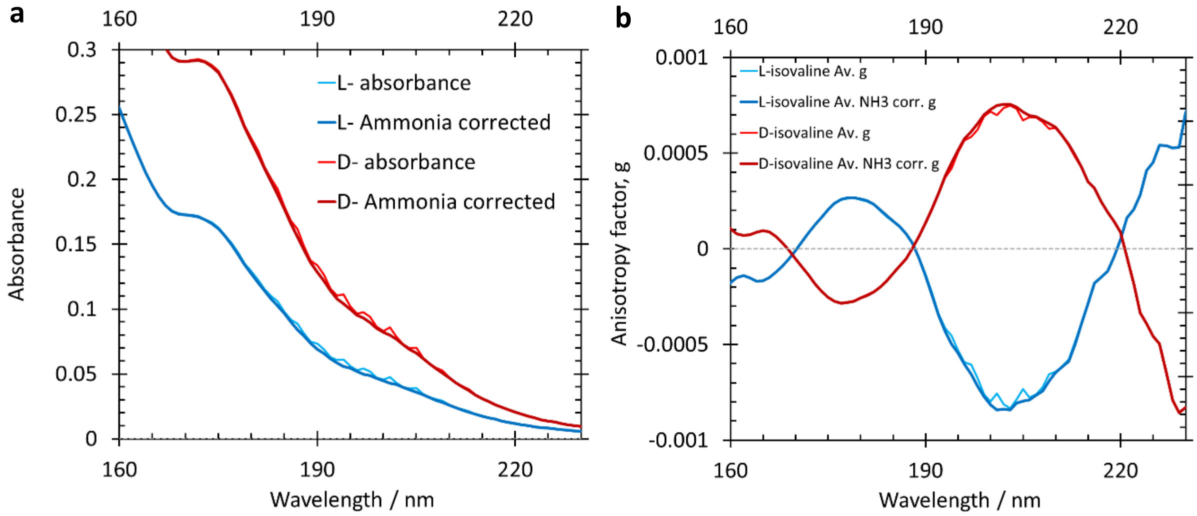

Supplementary Figure 11: Effect of the degradation product ammonia on the anisotropy spectrum of isovaline. **a**, Absorbance curves for L- (blue) and D- (red) isovaline, as measured (thin lines) and after correction for ammonia. **b**, Anisotropy spectra for L- (blue) and D- (red) isovaline, calculated using the absorbance as measured (thin lines) and after correction for ammonia.

Ammonia does not exhibit any spectral features above 215 nm, therefore, the impact on the highest measured anisotropy values for each amino acid is negligible as these generally occur at or above 220 nm. Although water does not have distinct spectral features which would allow subtraction from the measured absorbance spectrum, the conclusion is similar to the one for ammonia: since water

does not absorb above 185 nm, the highest measured anisotropy values are not affected by water vapour which might be present in the amino acid gas sample. Additionally, the overall cross section for absorption of water[31] is smaller than the cross section of ammonia[29] which, combined with the water degassing procedure described above in this section, further diminish the effect of water on the measured anisotropy spectrum.

Supplementary Note 4: Quantum chemical calculations

Three methods were evaluated: CAM-B3LYP[19], ωB97X-D[20] and MO6-2X[21] (**Supplementary Table 2**). Finally, seven low energy conformers were further considered for calculations. The calculated energies and conformer structures are summarized in **Supplementary Table 3**, where they can also be compared with literature values where these are available.

Experimental and calculated circular dichroism spectra of L-alanine were compared through the determination of the absolute value of local deviation as well as the root-mean-square deviation (RMSD). Overall best correlation was found using the M06-2X method (RMSD = 2.6) followed by the CAM B3LYP (RMSD = 3.2) and W97XD method (RMSD = 3.3). While major differences occur at higher energies for all three DFT methods due to inaccuracies of quantum chemical methods when approaching the ionization threshold as discussed in the main text, the absolute deviation from the experimental spectrum in the wavelength range of 215-250 nm is considerably lower for the calculations performed with M06-2X compared with the two other methods.

The experimental CD spectra of alanine shows a first negative band at 240 nm. The hybrid functional M06-2X with 54 % of HF exchange was the only method able to predict this negative band at 230 nm. The two other range-separated functionals CAM-B3LYP and ω97X-D lead to a shift in the negative band at 220 nm (**Supplementary Fig. 12**). Moreover, if conformer four is removed from the Boltzmann weighted CD spectra at T = 460 K using the hybrid functional M06-2X, the lowest lying CD band disappears completely indicating that conformer 4 dominates the CD sign and magnitude of the $n_{\mathrm{O}} \rightarrow \pi^{*}_{\mathrm{CO}}$ transition (**Supplementary Fig. 12e, f**).

Supplementary Table 2: Relative and total energies of the seven optimized low-energy conformers of L-alanine.

#	M062X aug-cc-pvQTZ[21]			CAMB3LYP aug-cc-pvQZ[19]			WB97-XD aug-cc-pvQZ[20]		
	E/hartree (a.u.)	ΔE/kJ mol^{-1}	Dihedral NCαCO	E/hartree (a.u.)	ΔE/kJ mol^{-1}	Dihedral NCαCO	E/hartree (a.u.)	ΔE/kJ mol^{-1}	Dihedral NCαCO
**1**	-323.689239	0.00	-13.7	-323.691216	0.00	-163.9	-323.725193	0.00	-18.1
**2**	-323.687461	4.67	-165.2	-323.690243	2.55	-170.4	-323.7224496	7.20	-16.1
**3**	-323.687364	4.92	164.8	-323.690103	2.92	168.4	-323.724372	2.16	166.6
**4**	-323.687287	5.12	-108.1	-323.689668	4.06	-96.5	-	-	-
**5**	-323.687164	5.45	146.6	-323.689401	4.77	142.5	-323.723354	4.83	145.0
**6**	-323.687016	5.84	-25.1	-323.68922	5.23	-25.2	-323.72314	5.40	-28.1
**7**	-323.686082	8.29	-135.6	-323.688221	7.86	-137.2	-323.721841	8.80	136.9

Supplementary Table 3: Conformation and dihedral angle *φ*(N–$C_α$–C=O) of MO6-2X[21] and coupled cluster CCSD(T)-F12[26] optimized conformers of L-alanine. The numbering of the low-energy conformers obtained in this study (1–7) are compared with previously reported conformers and experimental derived structural data.

	this study						*literature*	
**#**	**conformation**		***ΔE***[#]	***φ* (°)**[#]	***ΔE***[‡]	***φ* (°)**[‡]	**conformer**	***φ* (°)**
**1**	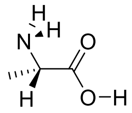	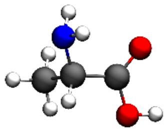	0.00	-13.7	0.00	-18.9	**1**[a], **I**[b, c]	-14[d], -16.6[e]
**2**	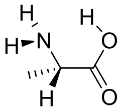	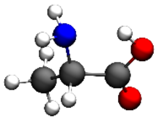	4.67	-165.2	2.72	-165.2	**2**[a], **IIB**[b], **III**[c]	
**3**	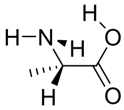	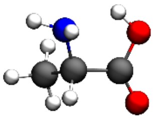	4.92	164.8	2.16	165.8	**3**[a], **IIA**[b], **II**[a]	167[d]
**4**	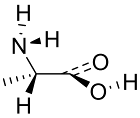	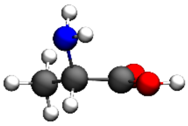	5.12	-108.1	5.36	-94.9	**5**[a], **IIIB**[b], **V**[c]	
**5**	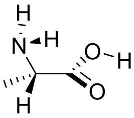	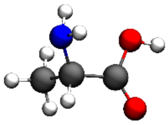	5.45	146.6	5.41	141.9	**4**[a], **IIIA**[b], **IV**[c]	
**6**	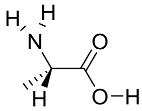	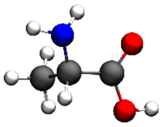	5.84	-25.1	4.75	-28.7	**6**[a], **IVA**[b]	
**7**	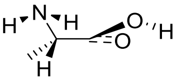	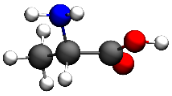	8.29	-135.6	7.24	-132.8	**8**[a], **VA**[b]	

[#]Electronic energies, Δ(*E* + *ZPE*) / kJ mol^{-1}, relative to conformer I and dihedral angles *φ* calculated at the MO6-2X level.

[‡]Electronic energies, Δ(*E* + *ZPE*) / kJ mol^{-1}, relative to conformer I and dihedral angles *φ* calculated at the CCSD(T)-F12 level.

[a] Data reported by Cao, M. *et al*[30].

[b] Data reported by Csaszar A.[23].

[c] Data reported by Godfrey, P. D. *et al.*[31].

[d] Experimentally derived structural data reported by Blanco, S. *et al.*[14]

[e] Experimentally derived structural data reported by Iijima, K. & Nakano, M.[32].

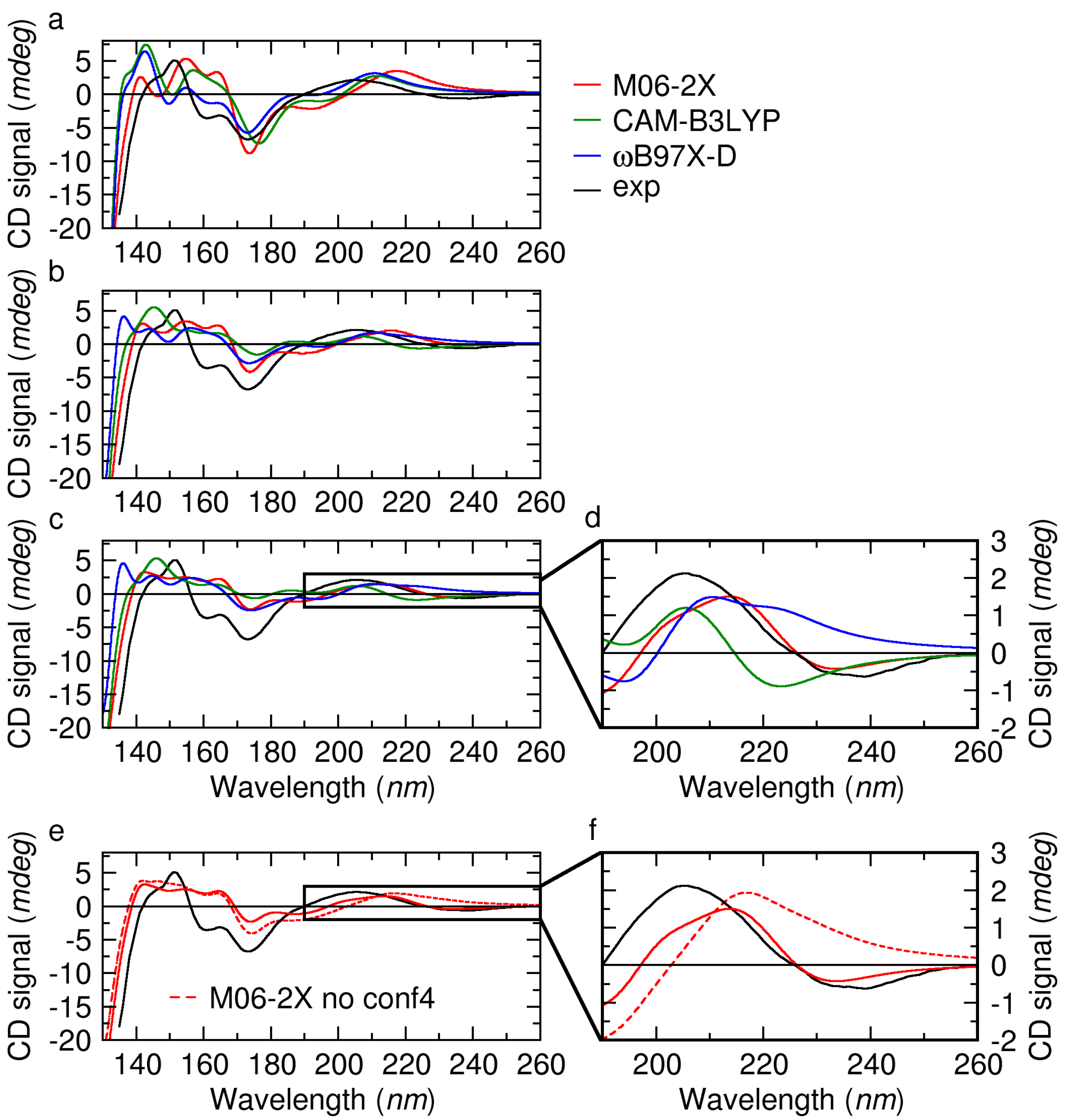

Supplementary Figure 12: Time-dependent density functional theory derived circular dichroism spectra of gas phase L-alanine. a, b, c, Comparison of experimental gas phase (black solid line) and theoretical CD spectra of alanine calculated for the first seven conformers at three different temperatures: **a**, $T = 0$ K; **b**, Boltzmann weighted at $T = 300$ K; and **c**, at $T = 460$ K. The theoretical spectra were obtained with M06-2X (red), CAM-B3LYP (green), and ωB97X-D (blue) by using the aug-cc-pvQZ basis set. The theoretical spectra have been obtained by summing rotatory strength weighted Gaussian curves with a constant half-width of 0.4 eV. **d**, the lowest lying CD band is best reproduced with M06-2X (red) at $T = 460$ K. **e**, **f** The lowest lying CD band at $T = 460$ K is dominated by conformer 4 revealed by the removal of conformer 4 from the Boltzmann weighted CD spectra (red dotted line).

Coupled cluster calculations

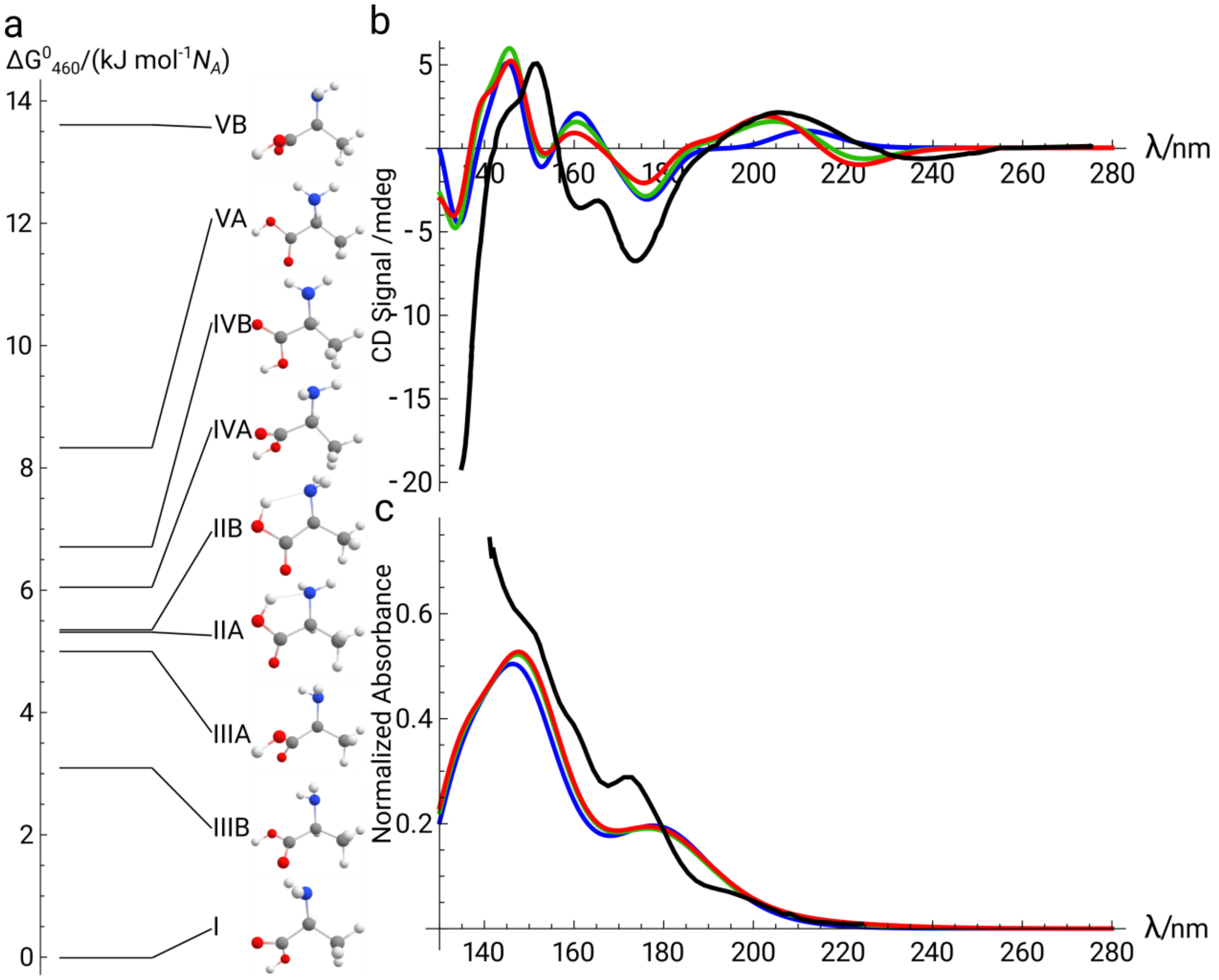

Supplementary Figure 13: Chiroptical gas phase spectra of L-alanine as computed on the coupled cluster level. **a**, Conformers of alanine with lowest standard Gibbs energy at T = 460 K and p = 0.03 mbar as determined on the CCSD(T)-F12/aug-cc-pVTZ//CCSD(T)-F12/aug-cc-pVDZ level of theory. **b**, **c**, comparison of experimental spectra of L-alanine (black line) and the various computed conformers (CC2/aug-cc-pVQZ) calculated at three temperatures, T = 0 K, lower energy (blue), Boltzmann weighted T = 300 K (green), and Boltzmann weighted T = 460 K (red). **b**, CD spectra, **c**, one-photon absorption spectra.

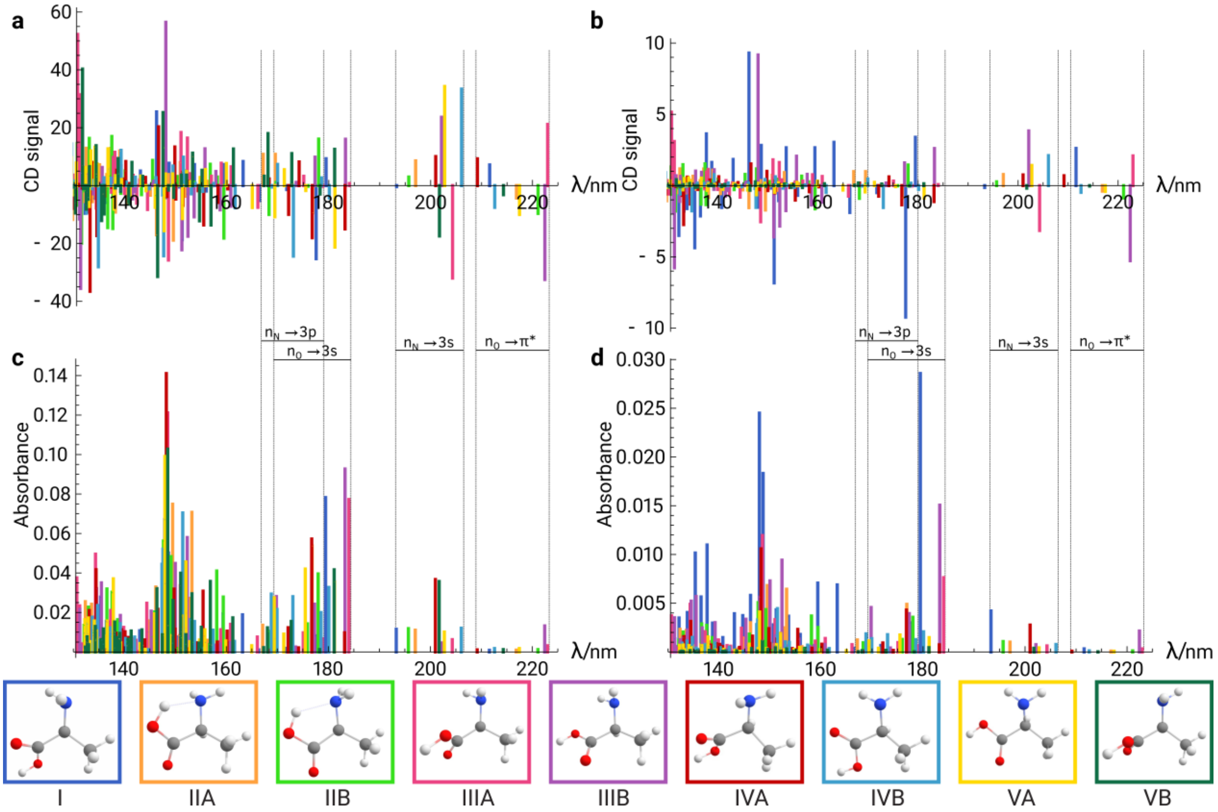

Supplementary Figure 14: Line spectra of low-energy conformers of L-alanine. **a**, **b**, CD spectra. **c**, **d**, one-photon absorption spectra. In **b** and **d**, the intensities are weighted using a Boltzmann distribution at 460 K with energies and frequencies from Table S4. The spectra in **a**, and **c**, are not weighted.

Supplementary Note 5: Polymerization test of alanine at elevated temperature

The sublimation of a commercial standard of enantiopure L-alanine was performed to determine if the dipeptide L-alanyl-L-alanine or *cyclo*-Ala-Ala is formed in the solid and/or in the gas phase during spectra recording, thereby contributing to the obtained CD and anisotropy spectra.

Approximately 20 mg of pure L-alanine was placed in a small glass container and heated under high vacuum in a sublimation chamber at 180 °C – the average temperature used to record the CD and anisotropy spectra of alanine enantiomers – for one hour, allowing sublimation and condensation of L-alanine onto a CaF_2 window. The non-sublimated L-alanine powder was removed from the glass container, weighed, and derivatized as *N*-ethyl chloroformate heptafluorobutyryl esters (ECHFBE). The sublimated L-alanine film was extracted with 5 x 50 µL milli-Q® water (TOC = 2 ppb) from the CaF_2 window, dried under a nitrogen flow, and derivatized as described above for the L-alanine residue. Both derivatized L-alanine samples, the non-sublimated residual powder and the sublimated L-alanine film were subsequently analysed using enantio-GC×GC-TOFMS.

The GC×GC-TOFMS analysis of a reference standard of L-alanyl-L-alanine results in two signals for the dipeptide and we consider its abundance to be the sum of these two signals. It was found that the L-alanine standard used for the sublimation tests, as well as for the CD and anisotropy measurements, contained small amounts of L-alanyl-L-alanine as revealed by GC×GC-TOFMS, due to contamination of the commercial standard. The L-alanyl-L-alanine abundance in the film (**Supplementary Fig. 15**) and in the residual powder from sublimation were compared to the L-alanyl-L-alanine abundance in the L-alanine reference standard. For this purpose, all samples were prepared at the same concentration

(4.5×10^{-4} M) and the area ratios of L-alanyl-L-alanine and L-alanine (A[L-Ala-L-Ala]/A[L-Ala]) were calculated to normalize the data. The resulting A[L-Ala-L-Ala]/A[L-Ala] ratios were very similar among all samples and resulted in values for the reference standard, the residual L-alanine powder and the sublimated L-alanine film of 0.0027, 0.0030 and 0.0025 respectively. This indicates that potential polymerization in the solid phase is negligible at these temperature and pressure conditions. The slight increase of the (A[L-Ala-L-Ala]/A[L-Ala]) ratio of the remaining L-alanine powder and the slight decrease of this ratio in the L-alanine film revealed that the film became slightly depleted in L-alanyl-L-alanine whereas the remaining powder became slightly enriched in the dipeptide. The lower volatility of the L-alanine dipeptide compared to L-alanine, making the dimer less prone to sublimation, most likely resulted in its accumulation in the remaining L-alanine powder. Based on a calibration curve of L-alanyl-L-alanine, we determined the percentage of L-alanyl-L-alanine in the commercial L-alanine standard to be less than 1 %, an amount that is not *a priori* expected to be measurable in gas phase circular dichroism spectroscopy and should therefore not make any important contribution to our CD and anisotropy spectra.

Moreover, we also searched for the cyclic diketopiperazine of alanine (*cyclo*-Ala-Ala, 3,6-dimethylpiperazine-2,5-dione) in the sublimated L-alanine film and the remaining L-alanine powder. Aliquots of the extracted sublimated L-alanine film and the remaining L-alanine powder, both at a concentration of 5×10^{-3} M, were analysed using HPLC coupled to an ELSD detector. No *cyclo*-Ala-Ala was detected. We determined the limit of detection of *cyclo*-Ala-Ala with the help of the commercial standard to be 5×10^{-5} M. Consequently, the potential formation of *cyclo*-Ala-Ala in sublimated alanine samples is at least below 1 %, a quantity that would not considerably alter the CD and anisotropy spectra.

In conclusion, we did not observe any substantial polymerization of L-alanine under the experimental conditions. Our gas phase CD experiments are therefore only very slightly altered by the amino acid degradation products and the intrinsic contamination of the commercial standards used. We do not expect any higher peptides to be formed in the gas phase.

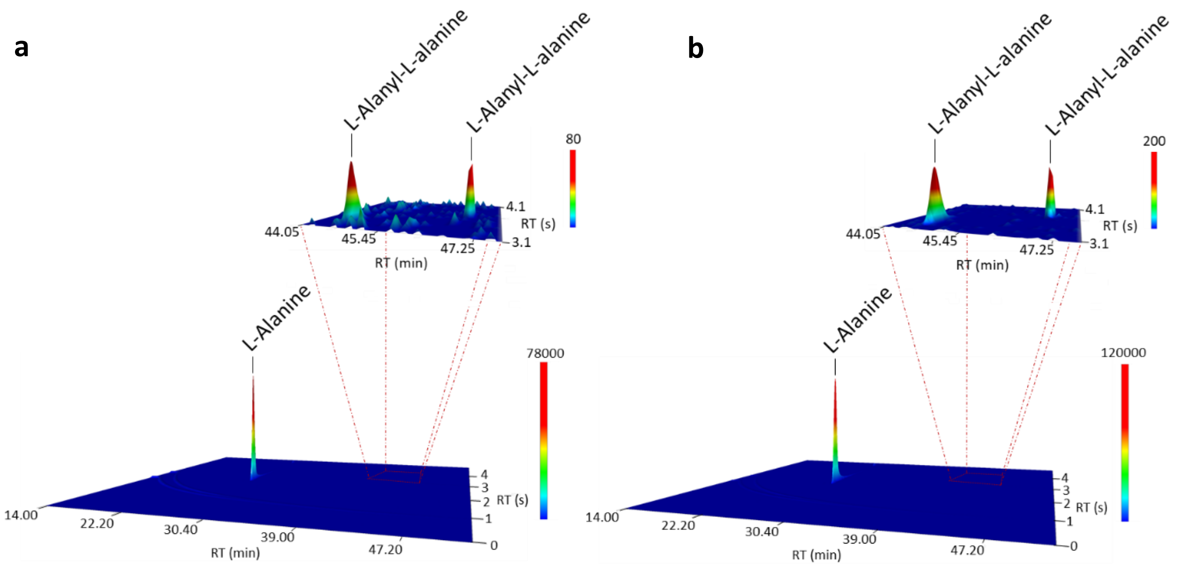

Supplementary Figure 15: Two-dimensional representation of the GC×GC-TOFMS analyses. **a,** Enantiopure L-alanine sample sublimated and condensed onto a CaF_2 window under vacuum at 180 °C (453.15 K). **b,** Analysis of enantiopure L-alanine and its impurities. The region of the L-alanyl-L-alanine is shown enlarged in the top of the figure and contains two signals that are summed to represent the abundance of the dipeptide.

Supplementary References:

[1] Ballard, R. E., Mason, S. F. & Vane, G. W. Circular dichroism of dissymmetric αβ-unsaturated ketones. *Discuss. Faraday Soc*. **35**, 43–47 (1963).

[2] Lambert, J., Compton, R. N. & Crawford, T. D. The optical activity of carvone: A theoretical and experimental investigation. *J. Chem. Phys*. **136**, 114512 (2012).

[3] Brint, P., Meshulam, E. & Gedanken, A. Excited electronic states of limonene: A circular dichroism and photoelectron spectroscopy study of d-limonene. *Chem. Phys. Lett*. **109,** 383–387 (1984).

[4] Gedanken, A. & Levy, M. New instrument for circular dichroism measurements in the vacuum ultraviolet. *Rev. Sci. Inst*. **48,** 1661 (1977).

[5] Gedanken, A., Duraisamy, M., Huang, J., Rachon, J. & Walborsky, M. Chiroptical properties of chiral olefins. *J. Am. Chem. Soc*. **110,** 4593–4599 (1988).

[6] Daly, S., Rosu, F. & Gabelica V. Mass-resolved electronic circular dichroism ion spectroscopy. *Science* **368,** 1465–1468 (2020).

[7] Miles, A. J., Hoffmann, S. V., Tao, Y., Janes, R. W. & Wallace, B. A. Synchrotron radiation circular dichroism (SRCD) spectroscopy: New beamlines and new applications in biology. *Spectroscopy* **21**, 245–255 (2007).

[8] Miles, A. J., Janes, R. W., Brown, A., Clarke, D. T., Sutherland, J. C., Tao, Y., Wallace, B. A. & Hoffmann, S. V. Light flux density threshold at which protein denaturation is induced by synchrotron radiation circular dichroism beamlines. *J. Synchrotr. Radiat*. **15**, 420–422 (2008).

[9] Duflot, D., Hoffmann, S. V., Jones, N. C. & Limao-Vieira, P. Synchrotron radiation UV-VUV photo-absorption of gas phase molecules. In *Radiation in Bioanalysis* (eds A. S. Pereira *et al.*), 43–81 (Spinger Nature, 2019).

[10] Svec, H. J. & Clyde, D. D. Vapor pressures of some α-amino acids. *J. Chem. Eng. Data* **10**, 151–152 (1965).

[11] Meinert, C., Hoffmann, S. V., Cassam-Chenaï, P., Evans, A. C., Giri, C., Nahon, L. & Meierhenrich, U. J. Photonenergy-controlled symmetry breaking with circularly polarized light. *Angew. Chem. Int. Ed*. **53**, 210–214 (2014).

[12] Meierhenrich, U., Filippi, J.-J., Meinert, C., Bredehöft, J. H., Takahashi, J.-I., Nahon, L., Jones, N. C. & Hoffmann, S. V. Circular dichroism of amino acids in the vacuum-ultraviolet region. *Angew. Chem. Int. Ed.* **49**, 7799–7802 (2010).

[13] Jochims, H. W. *et al.* Photoion mass spectrometry of five amino acids in the 6–22 eV photon energy range. *Chem. Phys*. **298**, 279–297 (2004).

[14] Blanco, S., Lesarri, A., Lopez, J. C. & Alonso J. L. The gas-phase structure of alanine. *J. Am. Chem. Soc.* **126**, 11675–11683 (2004).

[15] Kästner, J. Umbrella sampling. *WIREs Comput. Mol. Sci*. **1**, 932–942 (2011).

[16] Maier, J. A. *et al.* ff14SB: improving the accuracy of protein side chain and backbone parameters from ff99SB. *J. Chem. Theory Comput*. **11**, 3696–3713 (2015).

[17] Gaussian 16, Revision C.01, Frisch, M. J., Trucks G. W., Schlegel H. B., Scuseria G. E., Robb, M. A., Cheeseman, J. R., Scalmani, G., Barone, V., Petersson, G. A., Nakatsuji, H., Li, X., Caricato, M., Marenich, A. V., Bloino, J., Janesko, B. G., Gomperts, R., Mennucci, B., Hratchian, H. P., Ortiz, J. V., Izmaylov, A. F., Sonnenberg, J. L., Williams-Young, D., Ding, F., Lipparini, F., Egidi, F., Goings, J., Peng, B., Petrone, A., Henderson, T., Ranasinghe, D., Zakrzewski, V. G., Gao, J., Rega, N., Zheng, G., Liang, W., Hada, M., Ehara, M., Toyota, K., Fukuda, R., Hasegawa, J., Ishida, M., Nakajima, T., Honda, Y., Kitao, O., Nakai, H., Vreven, T., Throssell, K., Montgomery, J. A., Jr., Peralta, J. E., Ogliaro, F., Bearpark, M. J., Heyd, J. J., Brothers, E. N., Kudin, K. N., Staroverov, V. N., Keith, T. A., Kobayashi, R., Normand, J., Raghavachari, K., Rendell, A. P., Burant, J. C., Iyengar, S. S., Tomasi, J., Cossi, M., Millam, J. M., Klene, M., Adamo, C., Cammi, R., Ochterski, J. W., Martin, R. L., Morokuma, K., Farkas, O., Foresman, J. B., Fox, D. J. Gaussian, Inc. (Wallingford CT, 2016).

[18] Dunning Jr, T. H. Gaussian basis sets for use in correlated molecular calculations. I. The atoms boron through neon and hydrogen. *J. Chem. Phys.* **90**, 1007–1023 (1989).

[19] Yanai, T., Tew, D. P. & Handy, N. C. A new hybrid exchange–correlation functional using the Coulomb-attenuating method (CAM-B3LYP). *Chem. Phys. Lett*. **393**, 51–57 (2004).

[20] Chai, J.-D. & Head-Gordon, M. Long-range corrected hybrid density functionals with damped atom–atom dispersion corrections. *Phys. Chem. Chem. Phys.* **10**, 6615–6620 (2008).

[21] Zhao, Y. & Truhlar, D. G. The M06 suite of density functionals for main group thermochemistry, thermochemical kinetics, noncovalent interactions, excited states, and transition elements: two new functionals and systematic testing of four M06-class functionals and 12 other functionals. *Theor. Chem. Acc.* **120**, 215–241 (2008).

[22] Martin, R. L. Natural transition orbitals. *J. Chem. Phys*. **118**, 4775–4777 (2003).
[23] Csaszar, A. Conformers of gaseous α-alanine. *J. Phys. Chem*. **100**, 3541–3551 (1996).
[24] Werner, H.-J., Knowles, P. J., Manby, F. R. & Schütz, M. Molpro: a general-purpose quantum chemistry program package. *WIREs Comput Mol Sci* **2**, 242–253 (2012).
[25] Werner, H.-J., Knowles, P. J., Manby, F. R., Black, J. A., Doll, K., Heßelmann, A., Kats, D., Köhn, A., Korona, T., Kreplin, D. A., Ma, Q., Miller III, T. F., Mitrushchenkov, A., Peterson, K. A., Polyak, I., Rauhut, G. & Sibaev, M. *J. Chem. Phys*. **152**, 144107 (2020).
[26] MOLPRO, version, a package of ab initio programs, Werner, H.-J., Knowles, P. J., Knizia, G., Manby, F. R., Schütz, M. and others, see https://www.molpro.net
[27] TURBOMOLE V7.5 2020, a development of University of Karlsruhe and Forschungszentrum Karlsruhe GmbH, 1989-2007, TURBOMOLE GmbH, since 2007; available from https://www.turbomole.org.
[28] Mota, R., Parafita, R., Giuliani, A., Hubin-Franskin, M.-J., Lourenco, J. M. C., Garcia, G., Hoffmann, S. V., Mason, N. J., Ribeiro, P. A., Raposo, M. & Limao-Vieira, P. Water VUV electronic state spectroscopy by synchrotron radiation. *Chem. Phys. Lett*. **416**, 152–159 (2005).
[29] Limao-Vieira, P., Jones, N. C., Hoffmann, S. V., Duflot, D., Mendes, M., Lozano, A. I., da Silva, F. F., Garcia, G., Hoshino, M. & Tanaka, H. Revisiting the photoabsorption spectrum of NH_3 in the 5.4–10.8 eV energy region. *J. Chem. Phys*. **151**, 184302 (2019).
[30] Cao, M., Newton, S. Q., Pranata, J & Schaefer L. Ab inito conformational analysis of alanine. *J. Mol. Struct. THEOCHEM* **332**, 251–267 (1995).
[31] Godfrey, P. D., Firth, S., Hatherley, L. D., Brown, R. D. & Pierlot, A. P. Millimeter-wave spectroscopy of biomolecules: alanine. *J. Am. Chem. Soc.* **115**, 9687–9691 (1993).
[32] Iijima, K. & Nakano, M. Reinvestigation of molecular structure and conformation of gaseous L-alanine by joint analysis using electron diffraction data and rotational constants. *J. Mol. Struct*. **486**, 255−260 (1999).
