# Supplementary material for: Amino acid gas phase circular dichroism and implications for the origin of biomolecular asymmetry
Source: Nat Commun. 2022 Jan 26;13:502. doi: 10.1038/s41467-022-28184-0 (PMC8792022; doi:10.1038/s41467-022-28184-0)
Supplement: Supplementary file 3 — Description of Additional Supplementary Files [file 41467_2022_28184_MOESM3_ESM.docx]

Description of ADDITIONAL SUPPLEMENTARY FILES

Amino acid gas phase circular dichroism and implications for the origin of biomolecular asymmetry

Cornelia Meinert^1^*, Adrien D. Garcia^1^†, Jérémie Topin^1^†, Nykola C. Jones^2^, Mira Diekmann^3^, Robert Berger^3^, Laurent Nahon^4^, Søren V. Hoffmann^2^, Uwe J. Meierhenrich^1^*

**Supplementary Data files**

**File Name: Supplementary Data 1**

**Description:** Equilibrium structures of l-alanine conformers optimized with the program package Molpro on df-CCSD(T)-F12 with aug-cc-pVDZ-F12 basis set. Cartesian coordinates (x, y, z) are given in Å. Excel spreadsheet.

**File Name: Supplementary Data 2**

**Description:**Relative energies calculated with CCSD(T)-F12/aug-cc-pVTZ-F12 and harmonic vibrational wavenumbers calculated with df-CCSD(T)-F12/aug-cc-pVDZ-F12 of alanine conformers. Excel spreadsheet.
